# Selenobacteria-mediated Se transformation and uptake involving the unique genetic code

**DOI:** 10.3389/fpls.2024.1392355

**Published:** 2024-04-24

**Authors:** Qing Liao, Ao-Mei Li, Ying Xing, Pan-Xia Liang, Ze-Pu Jiang, Yong-Xian Liu, Dong-Liang Huang

**Affiliations:** ^1^ Guangxi Key Laboratory of Arable Land Conservation, Guangxi Academy of Agricultural Sciences, Nanning, China; ^2^ Guangxi Key Laboratory of Sugarcane Genetic Improvement, Guangxi Academy of Agricultural Sciences, Nanning, China; ^3^ Key Laboratory of Sugarcane Biotechnology and Genetic Improvement (Guangxi), Ministry of Agriculture and Rural Affairs, Guangxi Academy of Agricultural Sciences, Nanning, China

**Keywords:** selenobacteria, Se, biofortification, genome, Se activation

## Abstract

Selenium (Se) is a crucial micronutrient for human health. Plants are the primary source of Se for humans. Selenium in the soil serves as the primary source of Se for plants. The soil contains high total Se content in large areas in Guangxi, China. However, the available Se is low, hindering Se uptake by plants. Microorganisms play a pivotal role in the activation of Se in the soil, thereby enhancing its uptake by plants. In this study, selenobacteria were isolated from Se-rich soils in Guangxi. Then two selenobacteria strains, YLB1-6 and YLB2-1, representing the highest (30,000 μg/mL) and lowest (10,000 μg/mL) Se tolerance levels among the Se-tolerant bacteria, were selected for subsequent analysis. Although the two selenobacteria exhibited distinct effects, they can significantly transform Se species, resulting in a decrease in the soil residual Se (RES-Se) content while concurrently increasing the available Se (AVA-Se) content. Selenobacteria also enhance the transformation of Se valencies, with a significant increase observed in soluble Se^6+^ (SOL-Se^6+^). Additionally, selenobacteria can elevate the pH of acidic soil. Selenobacteria also promote the uptake of Se into plants. After treatment with YLB1-6 and YLB2-1, the Se content in the aboveground part of Chinese flowering cabbage increased by 1.96 times and 1.77 times, respectively, while the Se accumulation in the aboveground part of the plant significantly increased by 104.36% and 81.69%, respectively, compared to the control. Further whole-genome sequencing revealed the genetic difference between the two selenobacteria. Additionally, 46 and 38 candidate genes related to selenium utilization were identified from YLB1-6 and YLB2-1, respectively. This work accelerates our understanding of the potential molecular mechanism of Se biofortification by selenobacteria. It also provides microorganisms and gene targets for improving crop varieties or microorganisms to exploit the rich Se source in soil.

## 1 Introduction

Selenium (Se) is one of the 15 essential trace elements in the human body, regulating numerous physiological processes, earning the reputation of “element of miraculous life”. Se in the soil serves as the primary source of Se for humans. The pathway “soil-plant-human” is currently the most efficient and common Se biofortification way to supply Se for humans (Chilimba et al., 2012; Premarathna et al., 2012; Schiavon et al., 2020; Hossain et al., 2021).

Guangxi has a Se-rich soil area of 75,700 km^2^, making it the largest designated Se-rich soil region in China. Our team collected and analyzed 1,500 soil samples across Guangxi, revealing an average Se content in the soil of 0.55 mg/kg, with the highest recorded value being 2.29 mg/kg (Liu et al., 2015). This level far exceeds the average soil Se content in China, which is 0.29 mg/kg (China National Environmental Monitoring Station, 1990), signifying that the soil Se content in Guangxi is rich and high. However, in Guangxi, the majority of soils are acidic, and Se in such soils mainly forms insoluble oxides and hydroxides with iron, resulting in low available Se content (Geng, 2010). This hinders the utilization of the rich soil Se resources in Guangxi. Therefore, improving the available Se content in soil has become an urgent requirement to exploit soil Se resources not only in Guangxi but also globally.

The fractions and species of selenium (Se) in the soil are pivotal factors influencing Se availability (Shardendu et al., 2003). Microorganisms play a significant role in influencing the fractions, species, migration, and bioavailability of soil Se (Stolz et al., 2002, 2006; Valdez Barillas et al., 2011; Xu et al., 2017). They facilitate the conversion of Se fractions and species through processes such as oxidation, reduction, assimilation, and methylation (Nancharaiah and Lens, 2015), thereby enhancing the uptake of soil Se by plants (Ye et al., 2020; Yang et al., 2021). Consequently, Se biofortification by microorganisms emerges as a crucial approach for utilizing Se sources in the soil. Co-inoculation with selenobacteria strains, such as *Enterobacter* sp. B16, and the *arbuscular mycorrhizal* fungus *Glomus claroideum*, has been shown to significantly increase Se levels in wheat grains (Durán et al., 2013). Three bacterial strains isolated from corn rhizosphere soil exhibited a robust activating effect on Se in the soil, enhancing the soil’s water-soluble Se content (Long et al., 2017). A Se-tolerant endophyte, *Bacillus methylotrophicus* CSN-1, isolated from the leaves of *Cardamine hupingshanensis*, a plant accumulating super-high levels of Se, can convert selenite into SeCys2, enhancing plant uptake and transport of Se (Zhang et al., 2018). Inoculation of *Arbuscular mycorrhizal* fungi has been demonstrated to significantly promote the uptake of selenate or selenite by winter wheat (Luo et al., 2019).

In addition, bacteria harbor extensive genetic resources. The identification of genes associated with Se transformation in bacteria can offer potential targets for the improvement of bacteria or plants through molecular technologies, thereby enhancing Se utilization. Whole-genome sequencing facilitates the decryption of the genetic code of microorganisms, providing insights into candidate genes involved in Se utilization and contributing to a deeper understanding of the mechanisms underlying Se biofortification (Peng et al., 2016).

To enhance the genetic and microbial resources available for the biofortification of selenium (Se) in Se-rich soil, this study focused on the isolation and identification of highly Se-tolerant microorganisms from Se-rich soils in Guangxi. These microorganisms exhibit the capability to alter Se species and valencies in the soil, thereby facilitating Se uptake by plants. Additionally, the genomes of two distinct selenobacteria, characterized by divergent Se utilization capacities, were sequenced. This genomic information serves as a valuable resource for understanding the genetic basis of Se utilization. This work provides new microorganisms and genetic sources for the exploitation of rich Se depository in soil, and accelerate our understanding on the mechanism regarding Se biofortification.

## 2 Materials and methods

### 2.1 Culture media and reagents

Solid Media: Nutrient agar, Gause’s synthetic agar, potato dextrose agar were procured from Guangdong Huankai Microbial Sci&Tech Co., Ltd. (China).

Liquid Media: Prepared by following the formulations of the aforementioned solid culture media, excluding the agar component. Autoclaving was performed at 121°C, 15 PSI for 20 minutes before use.

PBS Buffer: Purchased from Shenggong Biotechnology (Shanghai) Co., Ltd. (China).

Na2SeO3 (AR 98%): Procured from Shandong Xiyachem Chemical Industry Co., Ltd. (China).

Se Solution (100 mg/mL): Weighed 22.35 g of Na2SeO3 (AR 98%), dissolved it in deionized water to make up 100 mL. The solution was filtered through a 0.22 μM sterile filter, stored in the dark for further use.

All other chemical reagents are domestically produced and of analytical grade.

### 2.2 Isolation of Se-tolerant microorganisms

Soil samples were collected from Se-rich areas in Guangxi, including Yongfu, Bama, Yulin, Guiping, and Teng county. Ten grams of soil sample were added to 90 mL of sterilized PBS buffer containing 10 glass beads, followed by shaking at 30°C and 200 rpm for 30 minutes, standing for 5 minutes, and collecting the suspension. Subsequently, the suspension was centrifuged at 5000 rpm for 15 minutes, and the sediment was resuspended in 10 mL of PBS buffer and stored at 4°C. Two mL of the above sample was added to 100 mL of liquid medium with a Se concentration of 100 μg/mL. Pure cultures of strains were obtained using gradient dilution and the streak plate method. The purified strains were then cultured in a liquid medium. Next, 5 μL of the 24-48 hour culture was inoculated into solid culture media with different gradient Se concentrations. Strains with poor Se tolerance were systematically eliminated to obtain strains with stronger Se tolerance.

### 2.3 Identification of high Se-tolerant bacteria

The 16S rDNA sequence of the highly Se-tolerant bacteria was determined by Nanning Guotuo Biotechnology Co., Ltd. (China). The acquired sequence was submitted to NCBI GenBank and compared to known sequences using the BLAST software. The phylogenetic tree was constructed using MEGA 5.0 software.

### 2.4 Pot experiment materials

Bacteria: two bacterial strains, *Bacillus cereus* YLB1-6 (Registration number in China Center for Type Culture Collection: M2020342, CCTCC NO: M2020342) and *Bacillus altitudinis* YLB2-1 (Registration number in Guangdong Microbial Culture Collection Center: 62524, GDMCC NO: 62524), were utilized for pot inoculation experiments. These strains were inoculated into a liquid medium and incubated at 37°C, 200 rpm for 24 hours. Following centrifugation at 5000 rpm for 10 minutes, bacterial cells were collected, washed twice with sterile 0.85% sodium chloride (NaCl), and then resuspended in sterile water to achieve a final bacterial suspension of approximately 1×10^8^ CFU/mL.

Soil: the soil selected for the pot experiment was obtained from Guiping City, Guangxi (109°56′59″ E, 23°18′36″ N). Characterized as lateritic red soil derived from granite, the basic physicochemical properties of the soil are as follows: total selenium content is 0.95 mg/kg, total nitrogen is 1.30 g/kg, total phosphorus is 0.736 g/kg, total potassium is 4.76 g/kg, alkali-soluble nitrogen is 129 mg/kg, readily available phosphorus is 46.0 mg/kg, readily available potassium is 82.6 mg/kg, organic matter is 16.0 g/kg, and the pH is 6.30. The soil underwent natural air-drying, grinding, and sieving through a 5 mm sieve. Before use, the soil was autoclaved at 121°C, 15 PSI for 30 minutes.

Plant: Chinese flowering cabbage was chosen as the plant for the experiment. Seeds were subjected to disinfection by immersion in 75% ethanol for 1 minute and 0.4% sodium hypochlorite for 2 minutes, followed by rinsing three times with sterile distilled water. Subsequently, the seeds were air-dried under aseptic conditions.

### 2.5 Pot experimental design

Three treatments were established, including a control group without bacteria (CK), YLB1-6 treatment, and YLB2-1 treatment, with three replicates for each treatment. Circular plastic pots, with an inner diameter of 18 cm and a height of 12 cm, were filled with 1.75 kg of soil. Following standard fertilization treatment, 0.2 g of urea and 0.4 g of potassium dihydrogen phosphate per kg of soil were added into each pot. Subsequently, 300 mL of distilled water was added to moisten the soil before sowing. In each pot, 10 Chinese flowering cabbage seeds were sown. The seedlings were thinned at the 4-leaves stage. and 6 seedlings, exhibiting similar growth vigor, were retained for each pot. Following this, 10 mL of bacterial suspension was inoculated into the rhizosphere of the plants, with an equal amount of sterile water serving as the control (CK). Sterile water was consistently employed throughout the pot experiment. The plants were cultivated in a glasshouse with natural light for a duration of 50 days before harvest. During the pot experiment, the temperature ranged between 20~35°C in the greenhouse, and soil moisture was maintained at approximately 70% of field capacity.

### 2.6 Plant sampling and measurement

The entire plants were harvested after 50 days of growth, washed with distilled water, and dried with absorbent paper. The aboveground and belowground parts of the plants were separated, and the fresh weight of each part was measured. Subsequently, the samples were heated at 105 °C for 30 minutes and dried at 60°C until a constant weight was achieved, then weighed. The dried samples were ground into fine powder, digested with HNO3-HClO4 (V:V=4:1), and reduced with 6 mol/L HCl. The Se content was measured using the hydride generation-atomic fluorescence spectroscopy method, following the “National Food Safety Standard-Determination of Selenium in Foods (GB 5009.93-2017)”.

Se Translocation factor (TF) and total Se amount were calculated based on the formulas:


TF=Se content in aboveground part (mg/kg)/Se content in underground part (mg/kg)

Total Se amount (µg/pot) = [Se content in aboveground parts (mg/kg) × the fresh weight of aboveground parts (g/pot) + Se content in underground parts (mg/kg) × the fresh weight of underground parts (g/pot)] ×1000. Here, 1000 is the conversion factor from mg to µg.

### 2.7 Soil sampling and measurement

After removing debris, the soil was air-dried. A portion was sieved through a 1mm mesh for pH determination, and another portion was sieved through a 100-mesh sieve for analyses of soil Se species and Se valencies. The pH was measured using the potentiometric method. Various Se species in the soil, including soluble Se (SOL-Se), exchangeable Se and carbonate-bound Se (EX-Se), iron (Fe)/manganese (Mn) oxide-bound Se (FMO-Se), organic matter-bound Se and elemental Se (OM-Se), and residual Se (RES-Se), were extracted using the continuous leaching method (Qu et al., 1997; Xing et al., 2018). The Se valencies, including Se^4+^, Se^6+^, and Se^2-^ in SOL-Se and EX-Se, were extracted following the method proposed by Wang et al. (2012). The Se species and valencies were measured using the hydride generation-atomic fluorescence spectroscopy method. The available Se in the soil (AVA-Se) is the sum of SOL-Se and EX-Se.

### 2.8 Genome sequencing

Genomic DNA was isolated from the cell suspension of the YLB1-6 and YLB 2-1 strains using the Wizard Genomic DNA Kit (Promega). DNA purification and concentration were estimated using Nanodrop and Qubit. DNA with high quality (OD260/280 = 1.8~2.0) was employed for further experiments. The genome was sequenced using PacBio SequelII platforms. The data obtained from the PacBio SequelII platform were utilized for bioinformatics analysis. The Flye software was employed for genome assembly, and Pilon was used to correct errors.

Glimmer version 3.02 was utilized for coding sequence (CDS) prediction, and the predicted CDSs were annotated from NR, Swiss-Prot, Pfam, Gene Ontology (GO), Clusters of Orthologous Groups (COG), and Kyoto Encyclopedia of Genes and Genomes (KEGG) databases using the NCBI-BLAST software, BLAST. For tRNA prediction and rRNA prediction, tRNA-scan-SE and Barrnap were employed, respectively. The prediction of SSR, Islands, and CRISPR sites was conducted using the trf and CRT software.

### 2.9 Statistical analysis

Data were analyzed by a one-way analysis of variance, and paired Duncan’s new multiple range test was performed using SPSS 17.0. All experiments were conducted in triplicate, and the values are presented as means ± standard errors. The level of significance was set at 0.05, and extreme significance was set at 0.01.

## 3 Results

### 3.1 Se-tolerant microorganisms were isolated from Se-rich soil in Guangxi

**Figure 1:**
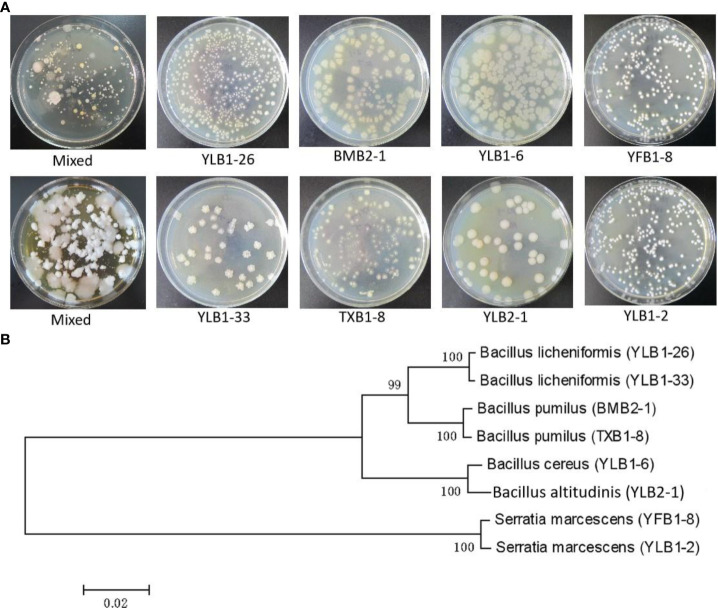
Isolation of Se-tolerant microorganisms. **(A)** Colony morphology of strains with high Se tolerance. **(B)** Phytogenetic analysis of Se-tolerant bacteria.

A total of 62 strains with distinct morphologies were isolated from soil samples collected across various Se-rich areas in Guangxi, encompassing 32 bacterial strains, 9 actinomycete strains, 10 fungal strains, and 11 yeast strains (**Figure 1A**). Through gradient Se concentration screening, bacteria exhibited the highest Se tolerance, reaching a concentration of 30,000 μg/mL, followed by fungi at 3,000 μg/mL, yeast at 1,500 μg/mL, and actinomycetes at 900 μg/mL. Among the 32 bacterial strains, 8 demonstrated tolerance to Se concentrations exceeding 10,000 μg/mL. Specifically, one strain could withstand 30,000 μg/mL (YLB1-6), two strains tolerated 29,000 μg/mL (YLB1-33, YLB1-26), two strains endured 20,000 μg/mL (YLB1-2, YFB1-8), two strains survived at 11,000 μg/mL (BMB2-1, TXB1-8), and one strain at 10,000 μg/mL (YLB2-1). The 16S rDNA sequencing identified these 8 bacterial strains as belonging to 5 species: *Bacillus licheniformis, Bacillus pumilus, Bacillus cereus, Bacillus altitudinis*, and *Serratia marcescens* (**Figure 1B**). These bacterial strains are classified as selenobacteria, which were designated in previous works (Acuña et al., 2013; Durán et al., 2015, 2018).

### 3.2 Selenobacteria enhance the transformation of Se in soil

To assess the impact of selenobacteria on Se biofortification, two selenobacteria strains, YLB1-6 and YLB2-1, representing the highest (30,000 μg/mL) and lowest (10,000 μg/mL) Se tolerance levels among the 8 Se-tolerant bacteria, were selected for a pot experiment. Inoculation with selenobacteria significantly reduced soil RES-Se content while increasing AVA-Se content. However, different selenobacteria exhibited varying effects on Se transformation. In comparison to CK, YLB1-6 decreased soil RES-Se by 13.18% while significantly increasing FMO-Se, SOL-Se, and EX-Se content. The latter two species resulted in a 4.22% increase in AVA-Se. YLB2-1 treatment led to a 9.80% reduction in RES-Se but a significant increase in OM-Se and SOL-Se content, as well as a 3.17% increase in AVA-Se, mainly contributed by SOL-Se (**Table 1**).

**Table 1:** Effect of selenobacteria on Se species in Se-riched soil (%).

Treatment	SOL-Se	EX-Se	AVA-Se	FMO-Se	OM-Se	RES-Se
CK	0.52 ± 0.06cB	10.08 ± 0.81b	10.60 ± 0.76bB	28.35 ± 1.92bB	16.47 ± 1.56bB	44.58 ± 3.90aA
YLB1-6	2.33 ± 0.45aA	12.49 ± 1.26a	14.82 ± 1.30aA	36.31 ± 1.25aA	17.47 ± 1.30bB	31.40 ± 3.63bB
YLB2-1	1.48 ± 0.13bAB	12.28 ± 0.47ab	13.77 ± 0.57aAB	29.55 ± 2.06bB	21.90 ± 0.64aA	34.78 ± 1.30bB

AVA-Se=SOL-Se+EX-Se. Uppercase and lowercase letters in the same column indicate significant difference at 0.01 and 0.05 levels, respectively.

**Figure 2:**
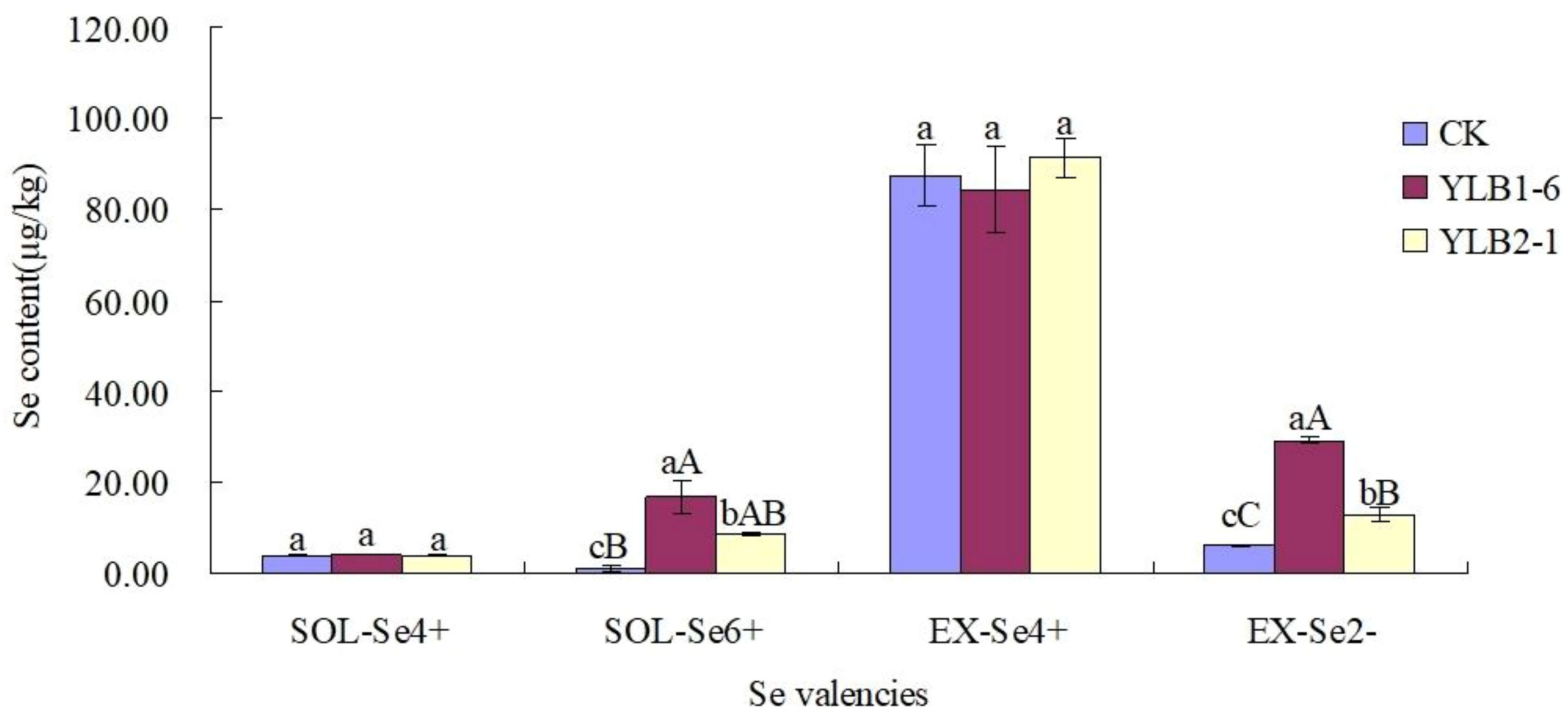
Effect of selenobacteria on Se valencies in Se-riched soil. Uppercase and lowercase letters in the same column indicate significant difference at 0.01 and 0.05 levels, respectively.

In the CK treatment, the soil Se content in different valencies exhibited the trend: EX-Se^4+^ > EX-Se^2-^ > SOL-Se^4+^ > SOL-Se^6+^. However, after selenobacteria inoculation, the soil Se content in different valencies showed the following trend: EX-Se^4+^ > EX-Se^2-^ > SOL-Se^6+^ > SOL-Se^4+^. These results indicate that after inoculation with selenobacteria, SOL-Se^6+^ significantly increased, but SOL-Se^4+^ did not show a significant change. Among the EX-Se species, EX-Se^4+^ did not change significantly, while EX-Se^2-^ increased extremely significantly (**Figure 2**).

### 3.3 Selenobacteria improve culture medium and soil pH value

pH is a critical factor influencing Se availability. In pot experiments, in comparison to CK, the pH increased by 4.20% in the YLB1-6 treatment and 1.59% in the YLB2-1 treatment. The pH elevation in the YLB1-6 treatment reached a highly significant level (**Table 2**).

**Table 2:** Effect of selenobacteria on soil pH value.

Treatment	pH
CK	6.27 ± 0.06bB
YLB1-6	6.54 ± 0.01aA
YLB2-1	6.37 ± 0.04bB

Uppercase and lowercase letters in the same column indicate significant difference at 0.01 and 0.05 levels, respectively.

### 3.4 Selenobacteria promote the uptake of Se into plant

**Figure 3:**
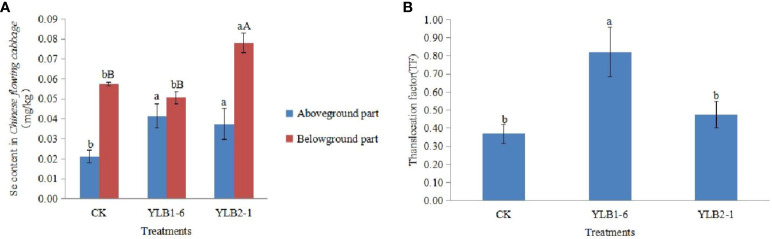
Effect of selenobacteria on Se uptake in Chinese Flowering Cabbage. **(A)** Se content in different parts of plants. **(B)** Se translocation factor in Chinese Flowering Cabbage. The uppercase and lowercase letters on the bar represent significant difference at 0.01 and 0.05 levels, respectively.

Following selenobacteria inoculation, the Se content in the aboveground part of Chinese flowering cabbage significantly exceeded that in the CK group. The Se content in the aboveground part after treatment with YLB1-6 and YLB2-1 was 1.96 times and 1.77 times that of the CK group, respectively, demonstrating that selenobacteria inoculation significantly enhances Se uptake in the aboveground part of the plant. However, a notable difference in the Se content in the underground part of the plant was observed between the two selenobacteria treatments. The Se content in the underground part of the YLB1-6 treatment did not differ significantly from that in the CK group, whereas the YLB2-1 treatment was significantly higher than that in the CK group, being 1.36 times that of the CK group. Additionally, the Se content in the underground part was higher than that in the aboveground part in all treatments. The Se content in the underground part of the CK group was 2.71 times that in the aboveground part, while in the YLB1-6 and YLB2-1 treatments, the Se content in the underground part was 1.22 times and 2.09 times that in the aboveground part, respectively (**Figure 3A**).

The Se Translocation Factor (TF) represents the ability of plants to transport Se from the underground part to the aboveground part. The TF for the CK treatment is 0.37, for the YLB1-6 treatment is 0.82, and for the YLB2-1 treatment is 0.48. Compared to CK, the TF of the YLB1-6 treatment increased by 123.14%, while the YLB2-1 treatment only increased by 29.29% (**Figure 3B**).

### 3.5 Selenobacteria increases Se accumulation but not biomass of Chinese flowering cabbage

**Figure 4:**
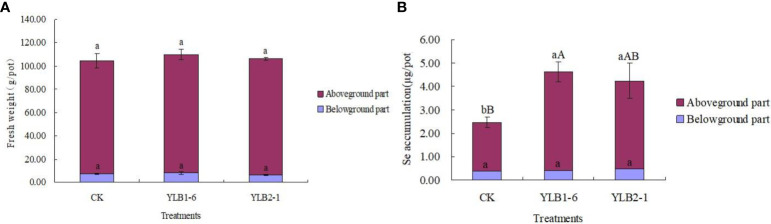
Effect of selenobacteria on the biomass **(A)** and Se accumulation **(B)** of *Chinese flowering cabbage*. The upcase and lowercase letters on the bar represent significant difference at 0.01 and 0.05 levels, respectively.

Even though the fresh weight of both the aboveground part and the underground part of the plant slightly increased with the bacteria treatments compared to CK, the differences, whether considering individual plant parts or the total fresh biomass, were not significant (**Figure 4A**). This suggests that inoculating selenobacteria does not improve the biomass of Chinese flowering cabbage.

Nevertheless, Se accumulation in the aboveground part of the plant significantly increased when treated with YLB1-6 and YLB2-1, with increases of 104.36% and 81.69% compared to CK, respectively. Although there were no significant differences in Se accumulation in the underground part of the plant between the different treatments, the total Se accumulation in the plant of YLB1-6 and YLB2-1 treatments showed increases of 87.35% and 71.83%, respectively, compared to CK. Additionally, YLB1-6 exhibited a better performance in Se accumulation than YLB2-1 did (**Figure 4B**).

### 3.6 Whole genome sequencing decipher the genetic code of selenobacteria

**Figure 5:**
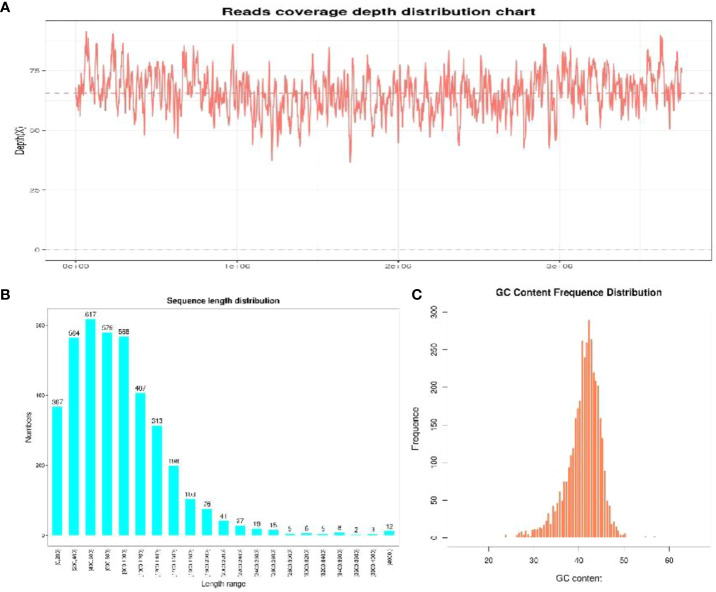
The sequencing quality of YLB1-6. **(A)** Reads coverage depth distribution chart. **(B)** Sequence length distribution. **(C)** GC content frequence distribution.

To unravel the genetic code of selenobacteria for Se biofortification, we conducted whole-genome sequencing of two selenobacteria strains with differing Se utilization abilities. For the YLB1-6 strain, characterized by higher Se utilization ability, the average genome depth was 35.32× (**Figure 5A**), ensuring complete genome coverage of 100%. The total gene length was 4,682,520 bp, with most genes falling within the 200 bp to 1400 bp range (**Figure 5B**), indicating the coding regions were fully sequenced, and the coding region occupied most proportion in the whole genome. The GC content of most CDS ranges from 30% to 40% (**Figure 5C**). Conversely, for the YLB2-5 strain with lower Se utilization ability, the average genome depth was 65.68×, also achieving complete genome coverage of 100%. The total gene length was 3,320,223 bp, with gene lengths mostly ranging from 200 bp to 1400 bp (**Supplementary Figure S1**), indicating comprehensive sequencing of coding regions dominating the genome. The GC content of most CDS ranged from 35% to 45% (**Supplementary Figure S1**).

**Figure 6:**
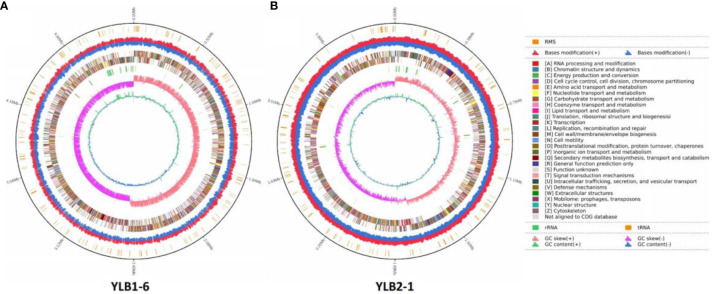
Landscape of the assembled YLB1-6 and YLB2-1 genome. From the outside, the first circle shows the RMS. The second circle shows the bases modification (+/-). The third circle shows the gene function classification. The fourth circle shows the GC content. The fifth circle shows the GC skew. **(A)** YLB1-6. **(B)** YLB2-1.

The genome of YLB1-6 comprises a 5,204,081 bp circular chromosome with an average G+C content of 35.39%, and a 425,392 bp circular plasmid DNA with a G+C content of 32.51%. Additionally, the genome encompasses 106 tRNA and 42 rRNA genes (**Table 3**). The chromosome houses 5,271 predicted genes (**Figure 6A**), while the plasmid genome contains 402 genes involved in RNA processing and modification, nucleotide transport and metabolism, amino acid transport and metabolism, signal transduction mechanisms, etc (**Supplementary Figure S2**).

**Table 3:** Genome characteristic of the two selenobacteria.

Characteristics	YLB1-6	YLB2-1
Genome size (bp)	5,629,473	3,757,945
GC content (%)	35.39	41.36
Topology Circular	Circular	Circular
Chromosome size (bp)	5,204,081	3,757,945
Plasmid size (bp)	425,392	/
Plasmid GC content (%)	32.51	/
Chromosome	1	1
Plasmid	1	
tRNA	106	81
rRNA	42	24
Gene (chromosome, plasmid)	5,673	3,935
TRF	397	109
SSR	2	1
Genomic islands	7	9
CRISPR	44	6
Prophage	3	2

In contrast, the genome of YLB2-1 consists of a 3,757,945 bp circular chromosome without a plasmid, featuring an average G+C content of 41.36%. The genome also includes 81 tRNA and 24 rRNA genes (**Table 3**). The chromosome contains 3,935 predicted genes, also engaged in RNA processing and modification, nucleotide transport and metabolism, amino acid transport and metabolism, signal transduction mechanisms (**Figure 6B**).

Moreover, utilizing Island Path-DIMOB, PHAST, and Minced software, the genome of YLB1-6 revealed 7 gene islands, 44 CRISPR (clustered regularly interspaced short palindromic repeats), and 3 prophages (**Table 3**). In parallel, the genome of YLB2-1 displayed 9 gene islands, 6 CRISPR, and 2 prophages (**Table 3**).

**Figure 7:**
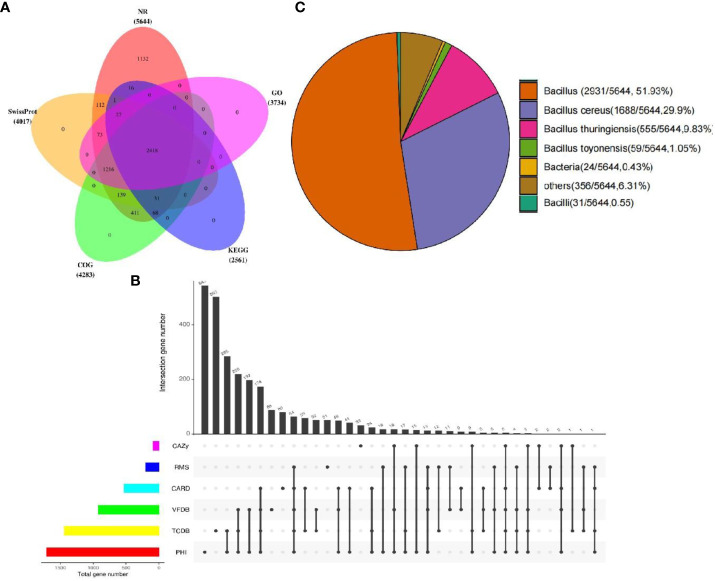
Annotated genes from YLB1-6. **(A)** The Venn diagram illustrates the distribution of genes across multiple databases. **(B)** Genes were annotated using five databases (CAZy, RMS, CARD, VFDB, TCDB, PHI), and common and unique genes were analyzed using UpSet plots (black indicates the presence of data at the point, grey indicates the absence of data at the point, and different points are connected to indicate intersection). **(C)** The distribution of species-annotated genes.

Among the 5,673 annotated genes from YLB1-6, 5,644 genes exhibited the best hit in the Non-redundant protein sequence database (Nr database). Additionally, 4,017, 4,283, 3,734, and 2,561 genes/proteins were annotated in Swissprot, COG, GO, and KEGG databases, respectively, with 2,418 genes common to all these databases (**Figure 7A**). Furthermore, 2,616 genes were annotated in special function databases, with 530 (9.34%) in CARD, 88 (1.55%) in CAZY, 1,707 (30.09%) in PHI, 920 (16.22%) in VFDB, 1,439 (25.37%) in TCDB, and 199 (3.51%) in RMA (**Figure 7B**). Of the 5,644 genes annotated in the Nr database, 2,931 (51.93%) were common genes in the genus Bacillus, with 1,688 (29.90%) showing significant similarity to those in *Bacillus cereus* (**Figure 7C**), as which YLB1-6 was confirmed by 16S rDNA sequencing.

For YLB2-1, out of the 3,935 annotated genes, 3,872 genes had the best hit in the Nr database. Furthermore, 3,296, 3,084, 2,948, and 2,163 genes were identified in Swissprot, COG, GO, and KEGG, respectively, and 2,076 genes were common to all these databases (**Supplementary Figure S3A**). In addition, 1,905 genes were annotated in special function databases, including 304 (7.73%) in CARD, 82 (2.08%) in CAZY, 1,309 (33.27%) in PHI, 655 (16.65%) in VFDB, 1,016 (25.82%) in TCDB, and 119 (3.02%) in RMA (**Supplementary Figure S3B**). Of the 3,872 genes mapped in the Nr database, 2,475 (63.92%) were common genes in the genus Bacillus, while 830 (21.43%) showed significant similarity to those in *Bacillus altitudinis* (**Supplementary Figure S3C**), as which YLB2-1 was identified by 16S rDNA sequencing.

### 3.7 Genes related to Se biofortification were screened from selenobacteria

Presently, numerous Se utilization-related genes have been either experimentally validated or computationally identified in diverse bacteria and archaea. Through a comprehensive examination of the YLB1-6 genome for known Se utilization-related genes, a total of 46 genes were discerned. These include peroxiredoxin (11), thioredoxin (15), thioredoxin reductase (5), methionine sulfoxide reductase A (1), seryl-tRNA synthetase (1), yqeB (1), yqeC (2), nitrite reductase (3), formate dehydrogenase (3), and arsenate reductase (4), with peroxiredoxin and thioredoxin being the most prevalent. These genes participate in diverse activities such as thioredoxin peroxidase activity, oxidoreductase activity, peroxiredoxin activity, glutathione peroxidase activity, peptide-methionine (S)-S-oxide reductase activity, serine-tRNA ligase activity, and formate dehydrogenase (NAD+) activity (**Table 4**). Their potential significance in Se utilization by the selenobacterial strain YLB1-6 is underscored.

**Table 4:** List of selenoproteins related genes in YLB1-6.

Gene_ID	Gene Annotation	E.C.number	MF_GO_term	Chromosome location
orf00834	Peroxiredoxin	EC:1.11.1.24	thioredoxin peroxidase activity	829636-830136
orf01000	Peroxiredoxin	EC:1.11.1.28	None	981904-982320
orf01870	Peroxiredoxin	–	oxidoreductase activity	1803231-1803806
orf02599	peroxiredoxin	–	peroxiredoxin activity	2535739-2536104
orf03627	Peroxiredoxin	–	disulfide oxidoreductase activity	3536596-3537150
orf03886	Peroxiredoxin	–	disulfide oxidoreductase activity	3778791-3779312
orf04551	Peroxiredoxin	–	None	4406725-4407204
orf04802	Peroxiredoxin	EC:1.11.1.24	thioredoxin peroxidase activity	4682581-4683036
orf04940	Peroxiredoxin	EC:1.11.1.24	peroxiredoxin activity	4833652-4834215
orf04802	Peroxiredoxin	EC:1.11.1.24	thioredoxin peroxidase activity	4682581-4683036
orf04941	NADH-dependent peroxiredoxin subunit F	EC:1.8.1.-	NADH dehydrogenase (ubiquinone) activity	4834230-4835756
orf00959	Thioredoxin	–	protein-disulfide reductase activity	942512-942826
orf02298	Thioredoxin	EC:1.20.4.4	arsenate reductase (thioredoxin) activity	2244092-2244496
orf03178	Thioredoxin	–	protein-disulfide reductase (NAD(P)) activity	3102040-3102354
orf03311	Thioredoxin	EC:1.11.1.9	glutathione peroxidase activity	3230333-3230815
orf03937	Thioredoxin	EC:1.8.4.8 1.8.4.10	phosphoadenylyl-sulfate reductase (thioredoxin) activity	3828404-3829108
orf04004	Thioredoxin	EC:1.17.4.1	ribonucleoside-diphosphate reductase activity	3883073-3884041
orf04156	Thioredoxin	hemoglobin	thioredoxin peroxidase activity	4025320-4025718
orf03974	Thioredoxin	–	–	3860804-3861358
orf04572	Thioredoxin	–	oxidoreductase activity	4424043-4424498
orf04888	Thioredoxin	–	–	4770415-4770729
orf04572	Thioredoxin	–	oxidoreductase activity	4424043-4424498
orf00484	Thioredoxin-like protein	–	protein-disulfide reductase activity	493746-494045
orf00775	Thioredoxin-like protein	–	protein-disulfide reductase activity	777071-777385
orf00790	Thioredoxin- like domain	–	–	792232-792558
orf00994	Thioredoxin- like domains	–	–	972877-973875
orf02589	Thioredoxin reductase	EC:1.5.3.1	oxidoreductase activity	2524662-2525897
orf03865	Thioredoxin reductase	EC:1.8.1.9	ferredoxin-NADP+ reductase activity	3759053-3760033
orf00343	Thioredoxin reductase	EC:1.8.1.9	thioredoxin-disulfide reductase activity	359319-360275
orf00550	Thioredoxin reductase	EC:1.18.1.2 1.19.1.1	ferredoxin-NADP+ reductase activity	556817-557806
orf04932	Thioredoxin reductase	EC:1.18.1.2 1.19.1.1	ferredoxin-NADP+ reductase activity	4825258-4826307
orf00051	Methionine sulfoxide reductase A	EC:1.8.4.11 1.8.4.12	peptide-methionine (S)-S-oxide reductase activity	53709-54674
orf05259	Seryl-tRNA synthetase	EC:6.1.1.11	serine-tRNA ligase activity	5184206-5185480
orf00622	yqeB	–	None	639828-640499
orf02123	yqeC	EC:1.1.1.44 1.1.1.343	phosphogluconate dehydrogenase activity	2065441-2066334
orf03216	yqeC	EC:1.1.1.44 1.1.1.343	phosphogluconate dehydrogenase activity	3138711-3139607
orf02591	Nitrite reductase	–	oxidoreductase activity	2526223-2526495
orf03934	Nitrite reductase	EC:1.7.7.1	ferredoxin-nitrite reductase activity	3824834-3826456
orf03934	Nitrite reductase	EC:1.7.7.1	ferredoxin-nitrite reductase activity	3824834-3826456
orf01915	Formate dehydrogenase	–	sulfurtransferase activity	1853161-1853958
orf04745	Formate dehydrogenase	EC:1.17.1.9	formate dehydrogenase (NAD+) activity	4611771-4614707
orf04746	Formate dehydrogenase	–	sulfurtransferase activity	4615008-4615814
orf02298	Arsenate reductase	–	cytoplasm	2244092-2244496
orf00481	Arsenate reductase	–	cytosol	492108-492473
orf02102	Arsenate reductase	–	cytosol	2043503-2043898
orf04164	Arsenate reductase	–	cytoplasm	4033716-4034111

Conversely, in strain YLB2-1 (**Table 5**), 38 Se utilization-related genes were identified, encompassing peroxiredoxin (12), thioredoxin (10), thioredoxin reductase (5), arsenate reductase (4), formate dehydrogenase assembly factor FdhD (1), glutaredoxin (1), methionine sulfoxide reductase (1), nitrite reductase (3), and seryl-tRNA synthetase (1). The distinct repertoire of Se-related genes in these two strains sheds light on the genetic underpinnings of their disparate Se-utilization abilities.

**Table 5:** List of selenoproteins related genes in YLB2-1.

Gene_ID	Gene Annotation	E.C.number	MF_GO_term	Chromosome location
orf01272	Lipoyl-dependent peroxiredoxin	EC:1.11.1.28	None	1267036-1267464
orf01274	Lipoyl-dependent peroxiredoxin	EC:1.11.1.28	None	1268106-1268525
orf00235	Peroxiredoxin	–	None	237999-238481
orf00867	Peroxiredoxin	EC:1.11.1.24	thioredoxin peroxidase activity	884365-884832
orf02141	Peroxiredoxin	–	disulfide oxidoreductase activity	2087897-2088427
orf02753	Peroxiredoxin	EC:1.11.1.24	thioredoxin peroxidase activity	2626798-2627298
orf03487	Peroxiredoxin	–	None	3327161-3327691
orf03882	Peroxiredoxin	–	None	3708946-3709470
orf00867	Peroxiredoxin	EC:1.11.1.24	thioredoxin peroxidase activity	884365-884832
orf01791	Peroxiredoxin	–	oxidoreductase activity	1752088-1752591
orf02753	Peroxiredoxin	EC:1.11.1.24	thioredoxin peroxidase activity	2626798-2627298
orf01388	Putative peroxiredoxin YkuU	–	peroxiredoxin activity	1367071-1367613
orf01389	thioredoxin	–	oxidoreductase activity	1367890-1368348
orf02682	Thioredoxin	–	oxidoreductase activity	2552794-2553108
orf02808	thioredoxin	–	protein-disulfide reductase activity	2680095-2680415
orf03122	thioredoxin	–	disulfide oxidoreductase activity	2963434-2963736
orf00226	Thioredoxin domain-containing protein	–	None	230813-232876
orf00830	Thioredoxin family protein	–	None	853409-853957
orf01794	Thioredoxin-like protein	–	None	1753000-1753245
orf00209	Thioredoxin-like protein SkfH	–	None	216834-217253
orf00488	Thioredoxin-like protein YdbP	–	protein-disulfide reductase activity	525361-525681
orf01791	Thioredoxin-like protein YneN	–	oxidoreductase activity	1752088-1752591
orf00688	Thioredoxin reductase	–	oxidoreductase activity	714865-715779
orf00814	Thioredoxin reductase	[EC:1.18.1.2 1.19.1.1]	ferredoxin-NADP+ reductase activity	838420-839460
orf02122	thioredoxin reductase	EC:1.8.1.9	ferredoxin-NADP+ reductase activity	2067780-2068811
orf03057	Thioredoxin reductase	–	ferredoxin-NADP+ reductase activity	2906043-2907041
orf03297	Thioredoxin reductase	EC:1.8.1.9	thioredoxin-disulfide reductase activity	3130668-3131651
orf01130	Arsenate reductase	–	None	1148457-1148852
orf02304	Arsenate reductase	–	None	2226370-2226750
orf03126	Arsenate reductase	EC:1.20.4.1	oxidoreductase activity	2964954-2965313
orf01807	Arsenate reductase (thioredoxin)	EC:1.20.4.4	arsenate reductase (thioredoxin) activity	1764421-1764840
orf03497	Formate dehydrogenase assembly factor FdhD	–	sulfurtransferase activity	3335177-3335965
orf03576	Glutaredoxin family protein		protein-disulfide reductase activity	3409641-3409895
orf01996	Methionine sulfoxide reductase	EC:1.8.4.11	peptide-methionine (S)-S-oxide reductase activity	1956071-1956604
orf01903	Nitrite reductase (NADH) large subunit	EC:1.7.1.15	nitrite reductase [NAD(P)H] activity	1862564-1864825
orf01901	Nitrite reductase [NAD(P)H]	EC:1.7.1.15	nitrite reductase NADPH activity	1857928-1860339
orf01900	Nitrite reductase small subunit NirD	EC:1.7.1.15	nitrite reductase NADPH activity	1857581-1857904
orf00014	Seryl-tRNA synthetase	EC:6.1.1.11	serine-tRNA ligase activity	22148-23422

## 4 Discussion

### 4.1 Isolation of selenobacteria

Selenium, a crucial trace element, plays a significant role in maintaining the health of both humans and animals (Rayman, 2012). Consequently, there is a growing concern regarding Se-enriched foods and the techniques employed in their preparation. In many areas, soil serves as a Se source with high total Se content. Harnessing the Se reservoir in soil presents a cost-effective and efficient means of developing natural food with elevated Se levels. However, while many Se-rich soils boast high total Se content, the available Se content tends to be low. Microorganisms are pivotal in Se bioactivation (Stolz et al., 2006; Valdez Barillas et al., 2011), making the screening of microorganisms with Se activation ability a crucial approach for effectively utilizing the abundant Se in soil (Jong et al., 2015; Zare et al., 2017; Ullah et al., 2020).

The screening of Se-activating bacteria, also referred to as selenobacteria, commonly involves exposure to high-Se concentrations, as documented in various studies (Prakash et al., 2010; Acuña et al., 2013; Durán et al., 2015, 2018). Under these elevated Se conditions, these bacteria demonstrate the capacity to adapt and accumulate higher levels of Se (Pusztahelyi et al., 2015). This screening method not only enhances the efficiency of Se enrichment but also leads to a significant increase in the production of biological Se. For instance, the Se-tolerant selenobacteria strain BSN313 demonstrated the ability to enrich Se up to 2,123 µg/g of dry weight (Ullah et al., 2020). In a study conducted in Enshi, China, three bacteria, *Acinetobacter baumannii, Bacillus licheniformis*, and *Bacillus subtilis* were isolated from the Se ore zone. Among these, *Acinetobacter* exhibited a high Se tolerance of up to 25,000 µg/mL, while the other two strains of Bacillus demonstrated the ability to tolerate Se concentrations exceeding 33,000 µg/mL (Peng et al., 2012).

In this study, liquid media containing a gradient of high Se content were employed for the initial screening of Se-tolerant microorganisms sourced from Se-rich soils in Guangxi, China. Through the utilization of gradient dilution spread plates, a total of 62 microorganisms with distinctive colony characteristics were isolated from diverse Se-rich soil samples. Among these, 32 were bacteria, 9 were actinomycetes, 10 were fungi, and 11 were yeast. A more detailed analysis of Se tolerance revealed that bacteria exhibited the highest Se tolerance, while actinomycetes displayed the lowest. Among the identified selenobacteria, *Bacillus cereus* YLB1-6 exhibited exceptional Se tolerance, with the ability to withstand a Se content of 30,000 µg/mL. This finding is particularly rare in Se-rich areas of Guangxi. Even the least tolerant strain, *Bacillus altitudinis* YLB 2-1, demonstrated a significant tolerance level of 10,000 µg/mL Se content. This work contributes valuable microbial resources for the exploration of abundant Se sources in soil.

### 4.2 Se transformation by selenobacteria

Se in soil is commonly classified into five species: SOL-Se, EX-Se, FMO-Se, OM-Se, and RES-Se (Wang et al., 2012). SOL-Se and EX-Se are readily absorbed and utilized by plants, representing available soil Se (Hu et al., 2014). The valencies of Se in soil include Se2-, Se0, Se4+, and Se6+ (Fernández-Martínez and Charlet, 2009). Se valencies that plants can take up and utilize include Se^4+^ and Se^6+^ (Qin et al., 2017), as well as some Se^2-^ forms, such as organic Se compounds like selenomethionine and selenocysteine (Guignardi and Schiavon, 2017).

Microorganisms play a pivotal role in transforming soil Se species and forming organic Se through metabolic processes involving Se transport, reduction, assimilation, oxidation, and methylation (Sarathchandra and Watkinson, 1981; Dowdle and Oremland, 1998; Winkel et al., 2012). The predominantly acidic red soils and lateritic soils in Se-rich areas of Guangxi, China, contain primarily selenite, which is prone to adsorption by iron oxides and clay minerals. However, microorganisms can transform adsorbed selenite into soluble Se for plant uptake (Shen and Zhou, 2011).

In this study, after inoculating selenobacteria, changes in Se species were observed, characterized by a decrease in RES-Se content and an increase in AVA-Se content in the soil. This indicates that selenobacteria can disrupt the equilibrium of Se species in the soil, promoting the transformation of residual Se into other Se species and thereby increasing the soil’s available Se content. Additionally, selenobacteria influence Se valency. After inoculation with selenobacteria, the soluble forms of SOL-Se^6+^ and EX-Se^2-^ significantly increased. This suggests that the elevated levels of SOL-Se^6+^ and EX-Se^2-^, following the inoculation of selenobacteria, contribute to the increased available Se content in Se-rich red soils.

### 4.3 Effect of selenobacteria on pH

Soil pH is a crucial factor influencing the available Se content in soil, typically increasing with rising soil pH of acidic soil. In our study, pH measurements were conducted on the soil after pot experiments, revealing a significant increase in soil pH with the application of YLB1-6. Changes in soil pH can impact the transformation of Se species, thereby influencing Se availability. After inoculation with selenobacteria, acidic soil pH and soluble Se content significantly increased.

The pH of soil is known to mediate the adsorption and desorption processes of Se with soil components such as Fe and Al ions (Goh and Lim, 2004). Our findings indicate that both selenobacteria could transform RES-Se, although the resulting Se forms differed. This variation may be attributed to differences in the metabolic by-products produced by distinct selenobacteria, leading to the formation of different Se species. Additionally, pH influences soil redox potential, microbial species, and activity, thereby affecting Se valency (Goh and Lim, 2004). This explains the observed increase in SOL-Se^6+^ and EX-Se^2-^ in the soil following the inoculation of selenobacteria.

### 4.4 Effect of Selenobacteria on Se uptake into plants

Inoculating microorganisms has been shown to enhance the uptake of soil Se by plants (Yang et al., 2021). Bacterial inoculation has increased Se content in the leaves of various crops such as wheat (Acuña et al., 2013), tea (Xu et al., 2020), and rice (Huang et al., 2020), as well as in the leaves, shoots, and seeds of Brassica juncea (Lampis et al., 2009; Yasin et al., 2015). Our study affirms that the inoculation of selenobacteria significantly increased Se content in the above-ground part of Chinese flowering cabbage compared to the control. Considering the Se transformation profile in soil, this increase is attributed to the conversion of residual Se into available Se by selenobacteria, leading to an augmentation in soluble Se^6+^ and exchangeable Se^2-^. These forms are readily absorbed by plants, a phenomenon also supported by Kikkert and Berkelaar (2013). Moreover, Se transport rates follow the order Se^6+^ > Se^2-^ > Se^4+^ (Chen, 2004; Jiang et al., 2015). Consequently, the Se^6+^ and Se^2-^ taken up by plants can be easily transported to the above-ground parts, resulting in Se accumulation in the aboveground part rather than the roots.

Furthermore, the YLB1-6 treatment exhibited higher Se accumulation in Chinese flowering cabbage than the YLB2-1 treatment, indicating that the YLB1-6 strain possesses a stronger capacity for activating soil Se. Consequently, more Se was taken up and transferred into the aboveground parts of plants in the YLB1-6 treatment, leading to a higher Se Translocation Factor (TF) than that of the YLB2-1 treatment. Currently, many studies aimed at enhancing Se uptake in plants involve the addition of exogenous Se (Huang et al., 2018; Xiong et al., 2019; Guo et al., 2020). Our work demonstrates that selenobacteria can effectively activate soil Se for plant uptake, providing alternative solutions for the exploitation of rich soil Se resources.

### 4.5 Genome analysis of selenobacteria

Deciphering the molecular mechanisms and identifying key genes involved in Se biofortification in selenobacteria is crucial for laying theoretical foundations and providing genetic resources for enhancing Se utilization in crops and microbial species through molecular technology. Whole-genome sequencing of microorganisms offers a comprehensive understanding of their genetic information, serving as a molecular basis for exploring the Se transformation mechanisms of selenobacteria and identifying critical genes for Se biofortification (Zhang et al., 2022). In this study, the genomes of two selenobacteria strains, YLB1-6 and YLB2-5, representing the highest and lowest Se tolerance within the bacteria group isolated, were extensively analyzed.

The genome of YLB1-6 revealed 5,673 genes, including 47 related to Se utilization, such as peroxiredoxin, thioredoxin, thioredoxin reductase, methionine sulfoxide reductase A, seryl-tRNA synthetase, yqeB, yqeC, nitrite reductase, formate dehydrogenase, and arsenate reductase. Meanwhile, YLB2-5, with lower Se utilization ability, exhibited 3,935 genes, including 38 associated with Se utilization. Notably, YLB2-5 lacked the key genes yqeB and yqeC, and the overall number of Se-related genes was lower compared to YLB1-6. This discrepancy likely contributes to the distinct Se utilization abilities of these two strains.

Peroxiredoxin (Prx) and methionine-S-sulfoxide reductase A (MsrA) are vital members of the selenoprotein family, playing crucial roles in Se transformation and utilization (Speckmann et al., 2016; Brigelius-Flohé and Flohé, 2017). Thioredoxin reductase is essential for selenite reduction in certain bacteria, and the selenite reduction via the Trx system is a pivotal early step for bacterial selenoprotein biosynthesis (Tamura et al., 2011; Shimizu et al., 2021). The tRNASec, charged with serine to yield seryl-tRNASec by canonical seryl-tRNA synthetase (SerRS), is involved in the production of selenoproteins. YqeB and YqeC are predicted to be associated with the utilization of Se-cofactor. Nitrite reductase enzymatically reduces selenite to elemental Se, a crucial step in Se metabolism (Kessi and Hanselmann, 2004; Tobe and Mihara, 2018; Huang et al., 2021).

The identification of 46 and 38 genes related to Se utilization in the genomes of YLB1-6 and YLB2-5, respectively, underscores their importance in Se biofortification. These genes represent potential targets for crop or microorganism variety improvement to enhance Se utilization.

## 5 Conclusion

**Figure 8:**
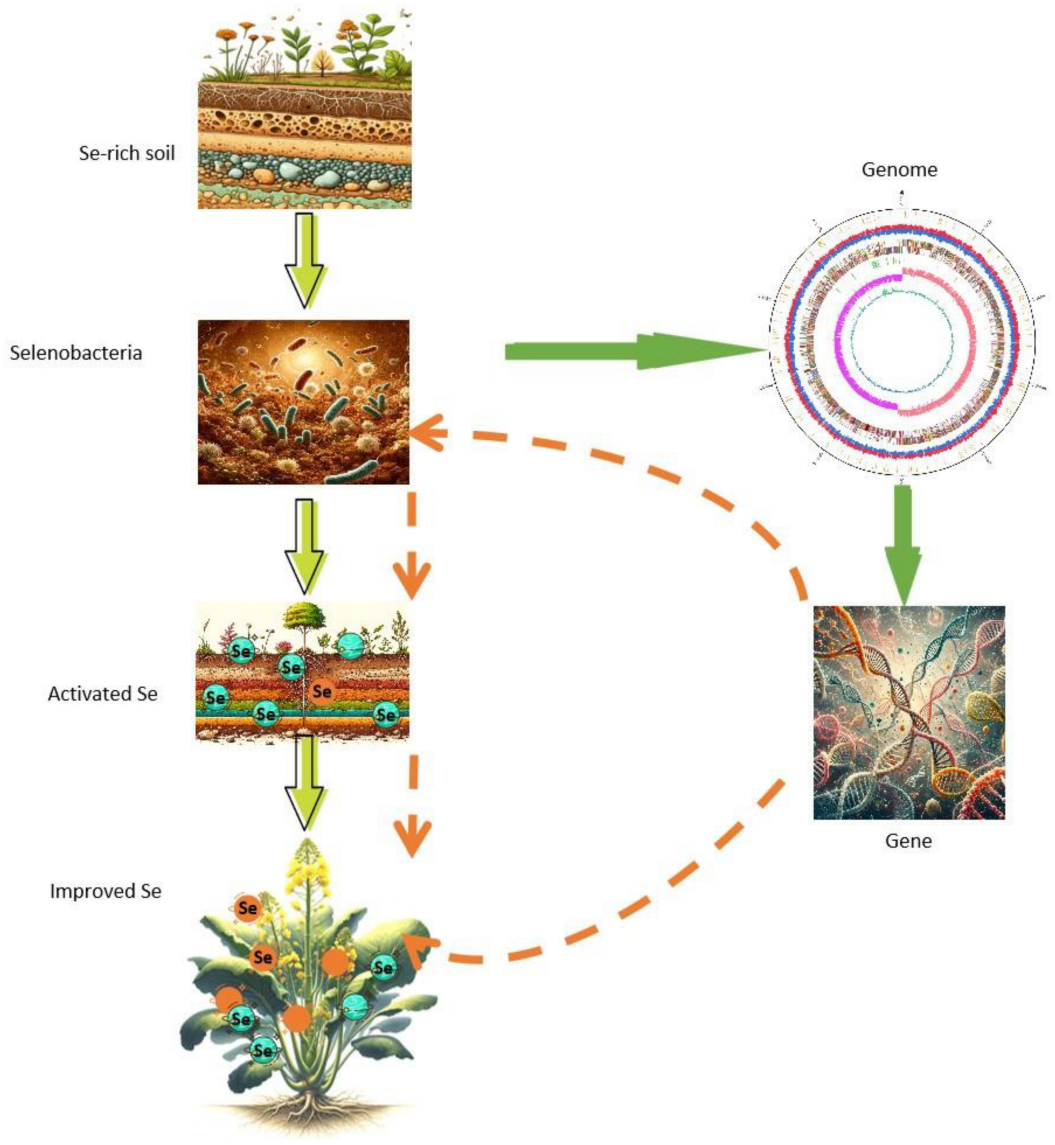
Isolation of selenobacteria and identification of candidate genes for Se fortification. Selenobacteria were isolated from selenium-rich soil, exhibiting the capability to activate selenium in the soil and facilitate its uptake into plants. The genomic analysis of selenobacteria unveiled candidate genes associated with selenium biofortification. These candidate genes can be leveraged to augment the selenobacteria’s proficiency in selenium activation, thereby enhancing the transformation of selenium into plants. Alternatively, these genes can be directly employed to enhance the plants’ capacity to uptake more selenium from the soil.

This study identified selenobacteria with a high Se-tolerant ability. Subsequent pot experiments demonstrated that these selenobacteria could transform the species and valencies of Se in the soil, facilitating Se uptake by plants. Whole-genome analysis unveiled the genetic elements of the two selenobacteria with contrasting Se utilization profiles and identified candidate genes related to Se utilization. This work significantly advances our understanding of the potential molecular mechanisms underlying Se biofortification by selenobacteria. Furthermore, it provides valuable microorganisms and gene targets for enhancing the Se utilization efficiency of crops or microorganisms, particularly for exploiting the rich Se sources in soil (**Figure 8**). This study also represents the first comprehensive genome dissection of selenobacteria.

## Data availability statement

The datasets presented in this study can be found in online repositories. The names of the repository/repositories and accession number(s) can be found below: https://www.cncb.ac.cn/, PRJCA021960.

## Author contributions

QL: Conceptualization, Formal analysis, Funding acquisition, Investigation, Methodology, Writing – original draft. A-ML: Conceptualization, Data curation, Investigation, Methodology, Software, Visualization, Writing – original draft. YX: Formal analysis, Investigation, Methodology, Writing – review & editing. P-XL: Formal analysis, Investigation, Methodology, Writing – review & editing. Z-PJ: Formal analysis, Methodology, Project administration, Supervision, Writing – review & editing. Y-XL: Conceptualization, Project administration, Supervision, Writing – review & editing. D-LH: Conceptualization, Supervision, Writing – review & editing, Project administration.

## Supplementary material

Supplementary Figure 1 The sequencing quality of YLB2-1. **(A)** Reads coverage depth distribution chart. **(B)** Sequence length distribution. **(C)** GC content frequence distribution.

Supplementary Figure 2 The plasmic genome in YLB 1-6. From the outside, the first circle shows the RMS. The second circle shows the bases modification (+/-). The third circle shows the gene function classification. The fourth circle shows the GC content. The fifth circle shows the GC skew.

Supplementary Figure 3 Annotated genes from YLB 2-1. **(A)** The Venn diagram of genes using multi databases. **(B)** Genes were annotated using five databases (CAZy, RMS, CARD, VFDB, TCDB, PHI), and the common and unique genes were analyzed using UpSet plots (black indicates the presence of data at the point, grey indicates the absence of data at the point, and different points are connected to indicate intersection). **(C)** The distribution of species annotated genes.
