# Supplementary figures and images for: Selenobacteria-mediated Se transformation and uptake involving the unique genetic code
Source: Front Plant Sci. 2024 Apr 24;15:1392355. doi: 10.3389/fpls.2024.1392355 (PMC11076775; doi:10.3389/fpls.2024.1392355)

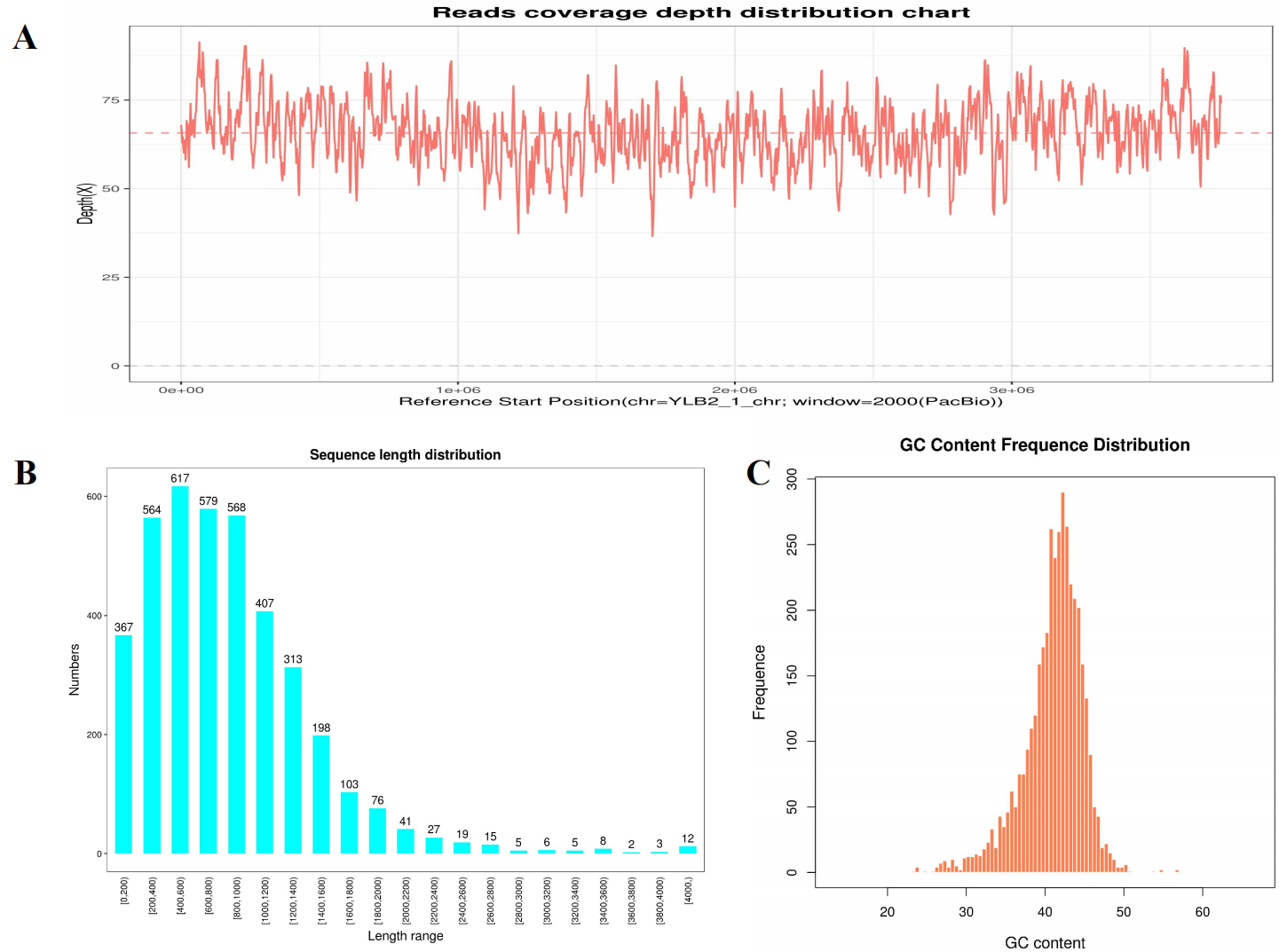

Supplement: Supplementary Figure 1 — The sequencing quality of YLB2-1. (A) Reads coverage depth distribution chart. (B) Sequence length distribution. (C) GC content frequence distribution. [file Image_1.tif]

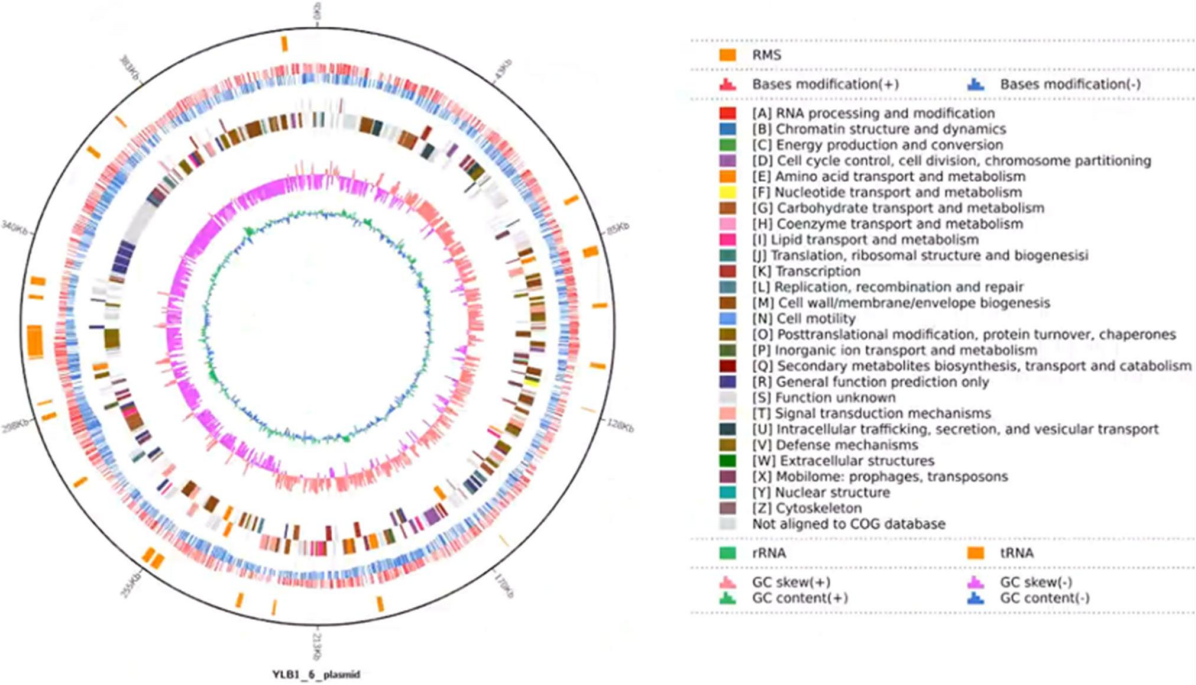

Supplement: Supplementary Figure 2 — The plasmic genome in YLB 1-6. From the outside, the first circle shows the RMS. The second circle shows the bases modification (+/-). The third circle shows the gene function classification. The fourth circle shows the GC content. The fifth circle shows the GC skew. [file Image_2.tif]

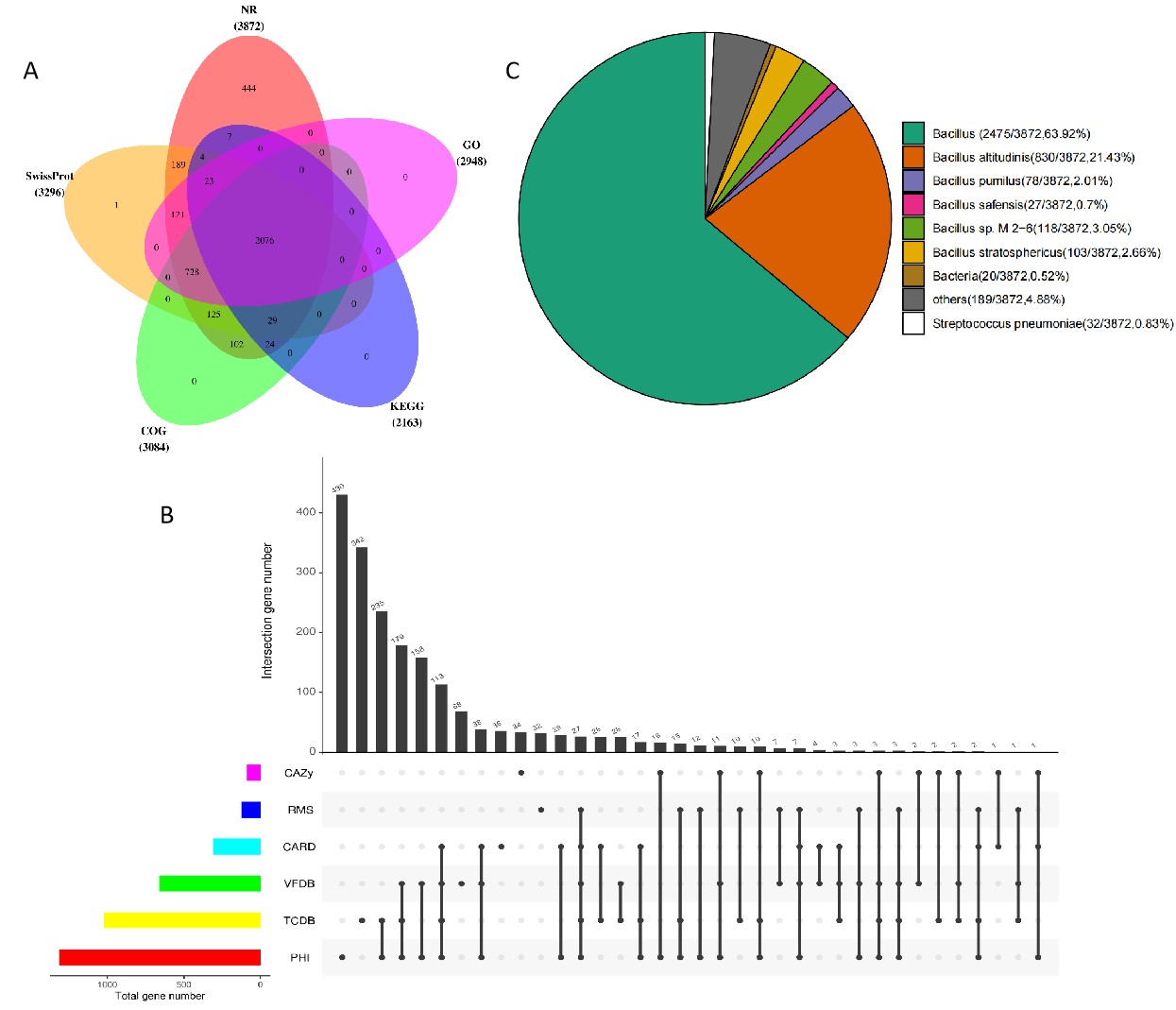

Supplement: Supplementary Figure 3 — Annotated genes from YLB 2-1. (A) The Venn diagram of genes using multi databases. (B) Genes were annotated using five databases (CAZy, RMS, CARD, VFDB, TCDB, PHI), and the common and unique genes were analyzed using UpSet plots (black indicates the presence of data at the point, grey indicates the absence of data at the point, and different points are connected to indicate intersection). (C) The distribution of species annotated genes. [file Image_3.jpeg]
